# Neck Dissection Reduces Risk of Recurrence in Early‐Stage Oral Tongue Cancer

**DOI:** 10.1002/hed.70017

**Published:** 2025-08-28

**Authors:** Jaclyn Lee, Tan Ding, Mae Wimbiscus, Dandan Liu, Melanie D. Hicks, Alexander J. Langerman, Robert J. Sinard, Kyle Mannion, Sarah L. Rohde, Eben L. Rosenthal, Michael C. Topf

**Affiliations:** ^1^ Department of Otolaryngology–Head and Neck Surgery Vanderbilt University Medical Center Nashville Tennessee USA; ^2^ Department of Biostatistics Vanderbilt University Medical Center Nashville Tennessee USA; ^3^ Vanderbilt University School of Medicine Nashville Tennessee USA; ^4^ Division of Head and Neck Surgery Vanderbilt University Medical Center Nashville Tennessee USA

**Keywords:** depth of invasion, early‐stage OTSCC, elective neck dissection, predictors of recurrence, tongue cancer

## Abstract

**Background:**

Patterns of failure in early‐stage oral tongue squamous cell carcinoma (OTSCC) managed with surgery alone remain understudied.

**Methods:**

Retrospective cohort study from 2000 to 2022 at a single tertiary care center evaluated pT1‐T2N0 OTSCC patients who underwent partial glossectomy with or without elective neck dissection (END), without any adjuvant therapy. Predictors of recurrence were analyzed via multivariate Cox proportional hazards model.

**Results:**

The study included 201 OTSCC patients (131 pT1 and 70 pT2). Eighty patients (40%) underwent glossectomy alone; 121 (60%) underwent glossectomy with END. At a median follow‐up of 56 months (IQR 23–82), 63 patients (31%) developed recurrence (21.4% local; 15.9% regional; 1% distant). Positive margins (HR 5.45 [1.9–15.4]), perineural invasion (HR 4.15 [1.8–9.9]), and increasing depth of invasion (DOI) (HR 1.32 [1.1–1.6]) were independently associated with recurrence. Upfront END reduced the risk of recurrence (HR 0.31 [95% CI: 0.15–0.6]), with benefit starting at tumor DOI 2 mm and greater.

**Conclusions:**

Upfront END decreases recurrence for pT1–T2N0 OTSCC treated with surgery alone starting at a DOI of 2 mm.

## 1 Introduction

Treatment for early‐stage oral tongue squamous cell carcinoma (OTSCC), i.e., T1‐T2N0, is typically surgical resection, with consideration of upfront elective neck dissection (END). Adjuvant radiation is recommended if there are adverse pathologic features, such as close margins, perineural invasion (PNI), lymphovascular invasion (LVI), or metastatic lymph nodes [1]. Despite the relatively favorable prognosis, recurrence rates following surgery alone for early‐stage OTSCC remain around 12%–40% [2, 3, 4, 5, 6].

The landmark D'Cruz et al. New England Journal of Medicine study published in 2015 demonstrated with prospective clinical trial data that patients with T1 or T2 oral cavity cancer benefit from END rather than a watchful waiting approach with therapeutic neck dissection as necessary [7]. Subgroup analysis showed patients with a 4 mm or greater depth of invasion (DOI) of the primary tumor had a greater overall survival benefit with upfront END [7]. Similarly, a retrospective review by Ganly et al. in 2013 that included 164 patients with pT1‐T2N0 oral tongue squamous cell carcinoma who underwent partial glossectomy and ipsilateral END without postoperative radiation from 1985 to 2005 also demonstrated tumor thickness of 4 mm or greater to be the only independent predictor of regional neck failure [8]. Most other studies have supported a DOI of 4 mm to be of clinical significance for increased risk of occult cervical nodes [9, 10], although some have found clinical significance as low as 3 mm [11, 12, 13] or even 2 mm [14, 15].

This current study aims to evaluate patterns of failure in patients with early‐stage oral tongue cancer (i.e., T1‐T2N0) treated with glossectomy, with or without END, and no adjuvant therapy within a single institution over a 22‐year period. In addition, we evaluate predictors of recurrence, and more specifically, whether END prevents recurrence.

## 2 Methods

This was a single‐institution, retrospective cohort study of all OTSCC patients treated at a single tertiary care center from January 2000 to December 2022. This study was approved by the Institutional Review Board at Vanderbilt University Medical Center (IRB #170768).

Main inclusion criteria were patients with pathologic T1 or T2 N0 OTSCC, treated with surgery alone. Exclusion criteria were pathologic T3–T4 tumors, presence of nodal disease on final pathology, presence of distant metastasis at presentation, adjuvant radiation therapy, adjuvant chemotherapy, previously treated head and neck cancer, concurrent secondary malignancy, and patients without any documented follow‐up records. Surgical treatment included primary tumor resection with or without upfront END. At our institution, ENDs for OTSCC are typically ipsilateral or bilateral level 1A, 1B, 2A, 2B, and 3. Data on neck levels dissected was not available for many patients due to the lengthy study period dating back to 2000. Patients that underwent neck dissection concurrently at the time of primary tumor resection, as well as those with an elective staged neck dissection, based on primary tumor pathology report within 4 weeks of glossectomy were included.

### 2.1 Study Measures and Outcomes

Patient demographics, disease characteristics, surgical details, and pathology report details were recorded. All patients were independently re‐staged based on their original surgical pathology report according to American Joint Commission on Cancer (AJCC) eighth edition criteria. Data collection was performed in May 2024. The main outcomes of this study were disease‐free survival and regional recurrence‐free survival, as well as predictors of recurrence. Secondary outcomes included local recurrence‐free survival and overall survival.

### 2.2 Statistical Analysis

Wilcoxon rank‐sum test and Pearson's Chi‐squared test were used to compare differences in demographic and disease characteristics between different patient groups. Multiple imputation with predictive mean matching was performed for patients with unavailable tumor DOI data (*N* = 18). Kaplan–Meier curves and log‐rank analyses were used to assess local recurrence‐free survival, regional recurrence‐free survival, and overall disease‐free survival. Multivariable logistic regression and Cox proportional hazards regression were used to assess the impact of patient, disease, and surgical variables associated with the risk of tumor recurrence (local, regional and/or distant) and patient overall survival. All testing was two‐sided, and statistical significance was defined as *p* ≤ 0.05. All analyses were performed using R version 4.3.2 (R Core Team, Vienna, Austria, 2023).

## 3 Results

### 3.1 Study Cohort

Over the 22‐year study period, 201 patients with T1‐T2N0 OTSCC treated with surgery alone without adjuvant therapy met inclusion criteria. Of these, 53.7% were male, with a median age of 60 years (IQR 50–69). Most of our patients were white (95%), and 112 patients (56.6%) were current or former tobacco users. Full details of patient and disease characteristics are shown in Table 1. Notably, upfront, ipsilateral END was performed in 121 patients (60.2%).

**TABLE 1:** Patient demographics and disease characteristics.

	Total cohort, *N* = 201
*Patient characteristics*	
Sex—Female, *N* (%)	93 (46.3%)
Age at surgery, years, median (IQR)	60 (50–69)
Race—White, *N* (%)	191 (95%)
Tobacco use history, *N* (%)	
Never smoker	86 (43.4%)
Former smoker	79 (39.9%)
Current smoker	33 (16.7%)
Alcohol use history, any, *N* (%)	116 (61.7%)
*Disease characteristics*	
Upfront elective neck dissection, *N* (%)	121 (60.2%)
Pathologic tumor staging, *N* (%)	
T1	131 (65.2%)
T2	70 (34.8%)
Pathologic N0 staging, *N* (%)	201 (100%)
DOI, ≥ 4 mm, *N* (%)	108 (53.4%)
3–3.9 mm	38 (18.9%)
2–2.9 mm	22 (10.9%)
< 2 mm	33 (16.4%)
Margins, negative, *N* (%)	201 (100%)
Primary tumor pathology, *N* (%)	
Perineural invasion	26 (12.9%)
Lymphovascular invasion	6 (3.0%)
Positive margins	8 (4%)
Histologic grade, *N* (%)	
1 Well‐differentiated	64 (32.7%)
2 Moderately‐differentiated	113 (57.7%)
3 Poorly‐differentiated	19 (9.7%)
*Recurrence*	
Disease recurrence, *N* (%)	63 (31.3%)
Local recurrence, *N* (%)	43 (21.4%)
Regional recurrence, *N* (%)	32 (15.9%)
Ipsilateral neck recurrence	29 (14.4%)
Contralateral neck recurrence	1 (0.5%)
Bilateral neck recurrence	2 (1%)
Distant recurrence, *N* (%)	2 (1%)
Follow‐up length, months (IQR)	56 (23–81)

Abbreviations: DOI, depth of invasion; IQR, interquartile range.

### 3.2 Pathologic Characteristics

Most patients (65.2%) were pT1, while 34.8% were pT2. Median DOI for the entire cohort was 4 mm (IQR 2–6). PNI was present in 26 patients (12.9%) and LVI was present in 6 patients (3%). All primary tumor resection margins were negative. All patients were pN0.

### 3.3 Patterns of Recurrence

Over a median follow‐up of 56 months (IQR 23–81), 63 patients (31.3%) developed recurrence. This included 43 patients (21.4%) with local recurrence, 32 patients (15.9%) with regional nodal recurrence, and 2 patients (1%) with distant recurrence. A total of 12 patients had concurrent local and regional recurrences. Of the regional recurrences, 29 were ipsilateral, 1 was contralateral, and 2 were bilateral.

Differences in patient and disease characteristics stratified by OTSCC recurrence are shown in Table 2. Patients who underwent upfront END had a recurrence rate of 23.8%; compared to 43.8% in those who had close observation of the neck (*p* = 0.002). This includes 9.1% with regional recurrence in the END group compared to 26.2% regional recurrence in the observation group (*p* = 0.001).

**TABLE 2:** Patient and disease characteristics, based on disease recurrence.

	No OTSCC recurrence, *N* = 138	Recurrent OTSCC, *N* = 63	*p*
*Patient characteristics*			
Sex—Female, *N* (%)	61 (44.2%)	32 (50.8%)	*0.39*
Age at surgery, years, median (IQR)	60 (52–69)	61 (45–69)	*0.59*
Race—White, *N* (%)	129 (93.4%)	62 (98.4%)	*0.13*
Tobacco use history, *N* (%)			*0.61*
Never smoker	56 (41.5%)	30 (47.6%)	
Former smoker	57 (42.2%)	22 (34.9%)	
Current smoker	22 (16.3%)	11 (17.5%)	
Alcohol use history, any, *N* (%)	78 (60%)	38 (65.5%)	*0.47*
*Disease characteristics*			
Upfront elective neck dissection, *N* (%)	93 (67.4%)	28 (44.4%)	**0.002**
Unilateral neck dissection	92 (66.7%)	28 (44.4%)	**0.008**
Bilateral neck dissection	1 (0.7%)	0	
Pathologic tumor staging, *N* (%)			*0.73*
T1	91 (65.9%)	40 (63.5%)	
T2	47 (34.1%)	23 (36.5%)	
Pathologic N staging, *N* (%)			
N0	100%	100%	*—*
DOI (mm), median (IQR)	4 (2–5)	4 (3–7)	*0.12*
Primary tumor pathology, *N* (%)			
Perineural invasion	15 (10.9%)	11 (17.5%)	*0.19*
Lymphovascular invasion	3 (2.2%)	3 (4.8%)	*0.32*
Histologic grade, *N* (%)			*0.62*
1 Well‐differentiated	47 (34.8%)	17 (27.9%)	
2 Moderately‐differentiated	75 (55.6%)	38 (62.3%)	
3 Poorly‐differentiated	13 (9.6%)	6 (9.8%)	
Follow‐up length, months (IQR)	49 (21–69)	74 (37–104)	< 0.001

Abbreviations: DOI, depth of invasion; IQR, interquartile range; OTSCC, oral tongue squamous cell carcinoma.

### 3.4 Survival Analysis

**FIGURE 1:**
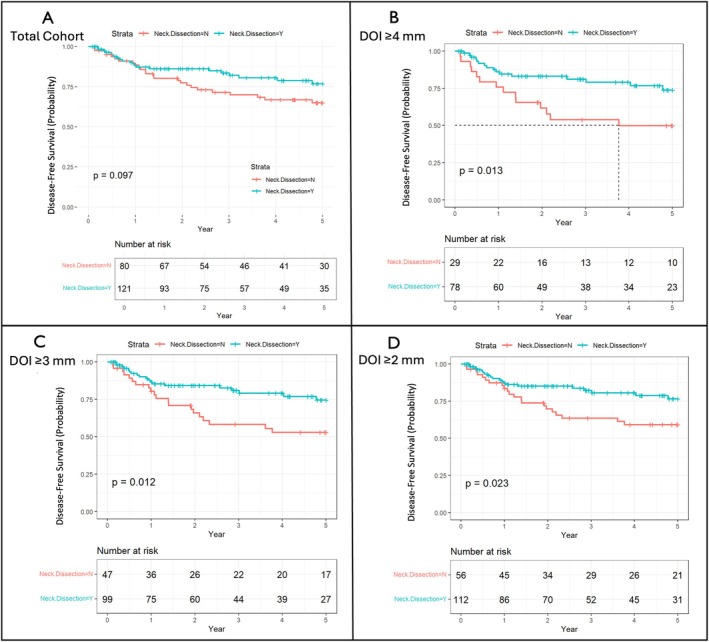
Kaplan–Meier curves for 5‐year disease‐free survival stratified by neck dissection. (A) Total cohort (*N* = 201), (B) patients with primary tumor DOI ≥ 4 (*N* = 108), (C) primary tumor DOI ≥ 3 (*N* = 146), and (D) primary tumor DOI ≥ 2 (*N* = 168). [Color figure can be viewed at wileyonlinelibrary.com]

The 5‐year rate of local disease‐free survival, local recurrence‐free survival, and regional recurrence‐free survival for our cohort was 78.6%, 88.6%, and 81.1%, respectively. For the entire cohort (*N* = 201), there was a trend toward improved disease‐free survival with upfront neck dissection, but this was not statistically significant (Figure 1). When we further divided patients into subgroups based on primary tumor DOI, a statistically significant benefit in disease‐free survival was seen based on the presence of upfront neck dissection. This was seen for a DOI cutoff as low as 2 mm and greater (*p* = 0.011; Figure 1). Using Kaplan–Meier curves and log‐rank tests, the DOI leading to maximum separation of disease‐free survival curves for our cohort showed 4 mm of DOI to be the optimal cutoff point. When examining overall survival, there were no differences in Kaplan–Meier curves stratified by neck dissection, regardless of tumor DOI subgroups (Supplemental Figure S1).

### 3.5 Predictors of Cancer Recurrence

Multivariate Cox proportional hazards regression evaluating predictors of disease recurrence is shown in Table 3. Upfront END was associated with a decreased risk of disease recurrence (hazards ratio [HR] = 0.31 [95% CI: 0.15–0.61], *p* < 0.001). Variables associated with an independently increased risk of recurrence were positive margins (HR = 5.45 [95% CI: 1.9–15.4], *p* = 0.001), DOI (HR = 1.32 [95% CI: 1.10–1.57], *p* = 0.002), and PNI (HR = 4.15 [95% CI: 1.76–9.88], *p* = 0.001).

**TABLE 3:** Multivariate cox proportional hazards ratio of risk factors related to disease recurrence.

	Hazards ratio (95% CI)	*p*
Age at surgery	0.99 (0.97–1.01)	0.36
pT2 stage (vs pT1)	0.77 (0.32–1.85)	0.56
Upfront elective neck dissection	0.31 (0.15–0.61)	< 0.0001
Primary tumor DOI	1.32 (1.10–1.57)	0.002
Primary tumor PNI	4.15 (1.76–9.88)	0.001
Tobacco use (current)	1.78 (0.77–4.17)	0.18
Year of surgery	3.19 (0.59–4.03)	0.35

Abbreviations: DOI, depth of invasion; PNI, perineural invasion.

On univariate Cox proportional hazards (Table 4), END showed a decreased risk of nodal recurrence specifically (HR = 0.39 [95% CI: 0.189–0.817], *p* = 0.012).

**TABLE 4:** Univariate cox proportional hazards ratio of disease recurrence with elective neck dissection as exposure variable.

	Estimate of coefficient	95% CI	*p*
Any recurrence	0.632	1.583, 0.3812	*0.074*
Local recurrence	0.783	0.426, 1.442	*0.43*
Regional nodal recurrence	0.393	0.189, 0.817	0.012
Distant recurrence	439 946 428	0, inf	*0.99*

## 4 Discussion

In this study, we evaluated risk factors associated with cancer recurrence in early‐stage oral tongue cancer (i.e., pT1‐T2N0) treated with surgery alone, with or without upfront elective neck dissection. Our survival analysis showed a statistically significant improvement in disease‐free survival with END for patients with primary tumor DOI of 2 mm and greater. This improvement in disease‐free survival was largely due to decreased regional recurrence. Factors independently associated with an increased risk of disease recurrence on multivariable Cox proportional hazards analysis were positive margins, primary tumor DOI, and presence of PNI. Upfront END was independently associated with a decreased risk of recurrence.

Understanding risk factors associated with recurrence in early‐stage oral tongue cancer drives complex postoperative tumor board discussions. Patients treated with surgical resection alone who did not receive END were independently at risk of OTSCC recurrence, especially in the untreated regional nodal basin. Our cohort had a 23% recurrence rate in those with upfront END, but 44% in those without (*p* = 0.002), which was largely driven by regional recurrence (9.1% vs. 26.2%; *p* = 0.001).

Prior studies have evaluated the role of END in early‐stage oral tongue cancer. D'Cruz et al. conducted a prospective randomized controlled trial of T1‐T2N0 oral cavity cancer patients, comparing END at the time of primary tumor resection with therapeutic neck dissection (i.e., after nodal recurrence), showing those with END had improved 3‐year overall survival and disease‐free survival [7]. While their survival analysis reported improved survival with upfront END starting with DOI of 4 mm and greater, our paper contributes to this existing literature by finding improved disease‐free survival with END beginning at primary tumor DOI of 2 mm and greater. Several retrospective cohort studies and systematic reviews have similarly found improved OTSCC outcomes and decreased recurrence rates with END [16, 17, 18]. Tongue cancer generally has a higher propensity for occult cervical nodal disease compared to other oral cavity cancers, given its extensive lymphatics network and occasional bilateral drainage [17]. The incidence of occult nodal metastasis for cN0 necks in cT1‐T2N0 OTSCC is reported to be as high as 46% [18, 19, 20]. An interesting finding of our study is the protective and therapeutic nature of END, despite our cohort being pathologically pN0. We believe that removal of high‐risk draining regional lymph nodes may offer a therapeutic benefit, beyond appropriate staging and adjuvant treatment guidance. Our findings could suggest that END at the time of initial primary resection removes occult metastatic lymph nodes that were unable to be detected on final pathology and that this removal decreases the risk of subsequent regional neck recurrence.

Our findings of improved survival with upfront END starting at a primary tumor DOI of 2 mm and greater is a smaller DOI than most other published studies—D'Cruz et al. [7], Ganly et al. [8], Kurokawa et al. [21], and Huang et al. [22] all reported DOI of 4 mm to be the cutoff of significance; Yuen et al. [11] reported clinical significance with 3 mm; and many studies have reported a DOI of 5 or 7 mm to be significant cutoffs [9, 10, 23, 24]. Few studies, such as Spiro et al. [14] and Shinn et al. [15] have reported clinical significance for occult nodal metastasis with 2 mm DOI. Based on our findings, we recommend consideration of END for primary tumors with DOI as low as 2 mm. This report offers an additional point of reference with regards to management of patients presenting with OTSCC. Our findings should complement current considerations regarding sentinel lymph node biopsy for OTSCC, which has early data showing equivalent survival outcomes as END for early‐stage oral cavity cancers and is currently undergoing phase II/III clinical trials across multiple institutions (NRG HN006) [25, 26].

Interestingly, in our study, the majority of nodal recurrences were ipsilateral; only three patients developed contralateral or bilateral neck recurrence, which is 9% of our regional recurrences. This is lower than the 39% reported by Ganly et al. [8], and lower than the 12%–28% described by other studies [6, 17, 27]. Another identified risk factor for recurrence in our study was PNI, supporting the role of adjuvant therapy in these patients. Unfortunately, in our retrospective cohort spanning three decades, we were unable to determine why these patients did not receive recommended adjuvant treatment.

We recognize several limitations of our study, including its retrospective nature, which inherently introduces bias, and single institution setting. Given our study spans the course of 22 years, we are not able to determine specific reasons for why some patients received END and some did not. However, when comparing between patients receiving END and those not, no differences were found in patient demographics or disease characteristics. Our multivariable cox proportional hazards also adjusted for year of surgery as a potential confounder, showing an independent survival benefit with END, regardless of year of surgery. Our Figure S2 shows a depiction of the proportions of END changes over time, based on year of surgery. The role and influence of adjuvant therapy on outcomes were outside of the scope of this study and are another limitation. Given the length of the study and practice guidelines that have changed over time, some patients in our cohort had adverse features on final pathology report and should have received adjuvant therapy; however, we cannot determine the reason for omission. Our goal with this paper is to provide a comprehensive assessment of all patients evaluated at our institution over the course of two decades, but we recognize this is a limitation that introduces heterogeneity in our cohort.

Additionally, a small minority of primary tumor specimens (*N* = 18) did not have a DOI measurement reported on final surgical pathology, which required data imputation of DOI via multivariate chained equations. While the distribution of the imputed variable set exactly matched that of the original dataset, this could undoubtedly introduce elements of uncertainty. It should also be noted that a significant proportion of our cohort had DOI ≥ 4 mm (53%), both in the END and no END group, which has the potential of skewing our findings. Given the length of our study, the original pathology slides were not available for independent re‐review. As such, certain pathological features, such as worse pattern of invasion, PNI size categories, and primary tumor margin distance, were not examined, which have all been reported to be important prognosticators [10, 21]. As a whole, our study offers an important observation to help guide treatment and management strategies for oral tongue cancer. Our findings would be further strengthened by future structured studies and prospective trials evaluating upfront END in OTSCC patients stratified by tumor DOI as low as 2 mm, though we recognize the difficulty in conducting a prospective clinical trial of that magnitude.

## 5 Conclusions

Early‐stage oral tongue cancer managed with surgery alone has acceptable locoregional control, which is improved with upfront END at a primary tumor DOI as low as 2 mm or greater. Therefore, END should be strongly considered in cT1‐T2N0 OTSCC patients. DOI and PNI were associated with increased risk of recurrence, which supports current recommendations for adjuvant radiation therapy in these patients. Our findings would be further strengthened by a large multi‐institutional cohort study to support our conclusions.

## Conflicts of Interest

The authors declare no conflicts of interest.

## Supporting information


**Figure S1:** Kaplan–Meier curves for 5‐year overall survival stratified by neck dissection. (A) total cohort (*N* = 200), (B) patients with primary tumor DOI ≥ 4 mm (*N* = 108), (C) tumor DOI ≥ 3 mm (*N* = 146), and (D) tumor DOI ≥ 2 mm (*N* = 167).
**Figure S2:** Proportion of elective neck dissection by year of surgery.

## Data Availability

The data that support the findings of this study are available on request from the corresponding author. The data are not publicly available due to privacy or ethical restrictions.
