# Supplementary material for: Neck Dissection Reduces Risk of Recurrence in Early‐Stage Oral Tongue Cancer
Source: Head Neck. 2025 Aug 28;48(2):339–45. doi: 10.1002/hed.70017 (PMC12796990; doi:10.1002/hed.70017)

**Supplemental Figures**

**Figure S1.** Kaplan-Meier Curves for 5-Year Overall Survival Stratified by Neck Dissection. **A:** total cohort (N = 200), **B:** patients with primary tumor DOI ≥ 4 mm (N = 108), **C:** tumor DOI ≥ 3 mm (N = 146), and **D:** tumor DOI ≥ 2 mm (N = 167).Click or tap here to enter text.


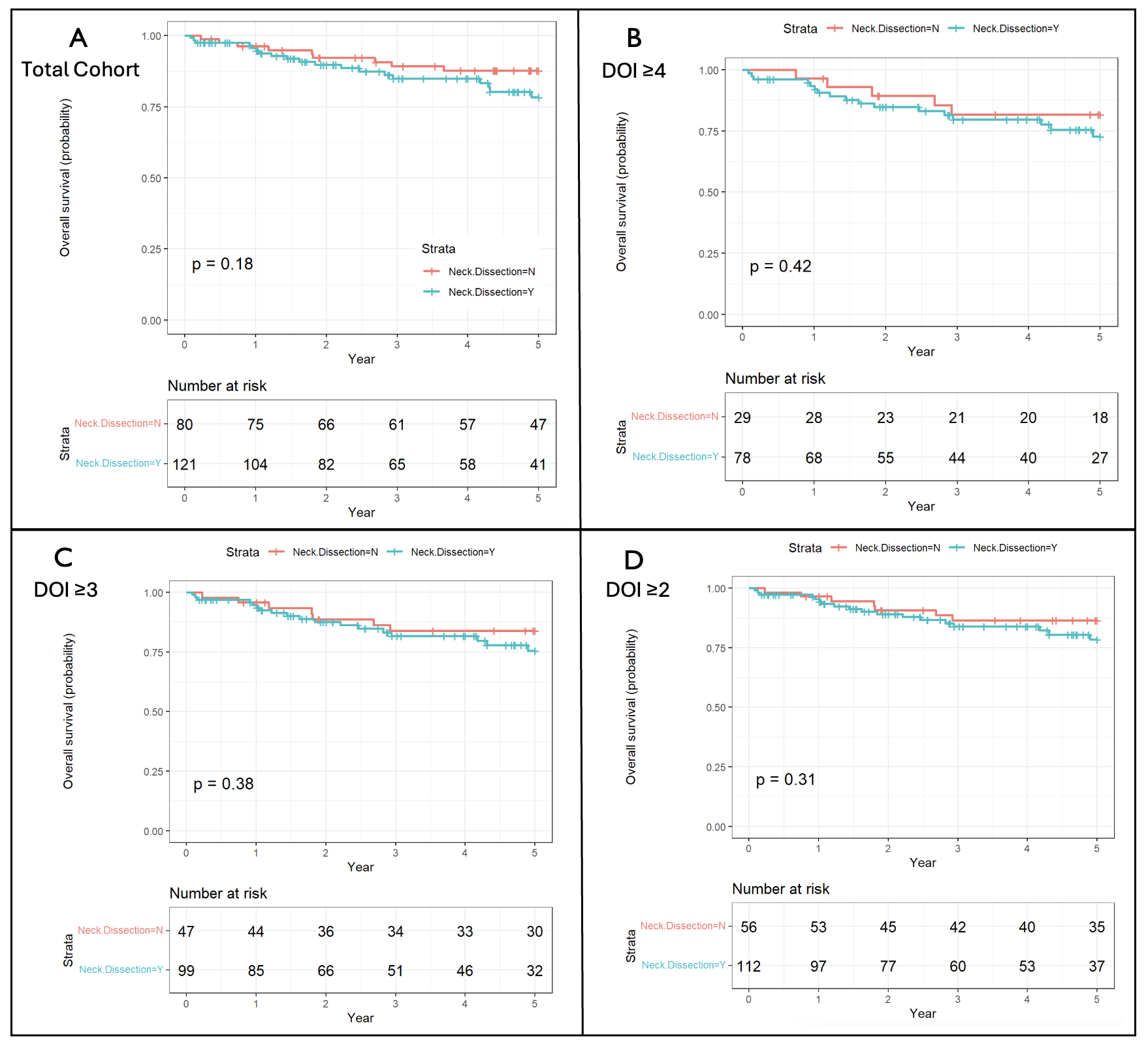


**Figure S2.** Proportion of Elective Neck Dissection by Year of Surgery.


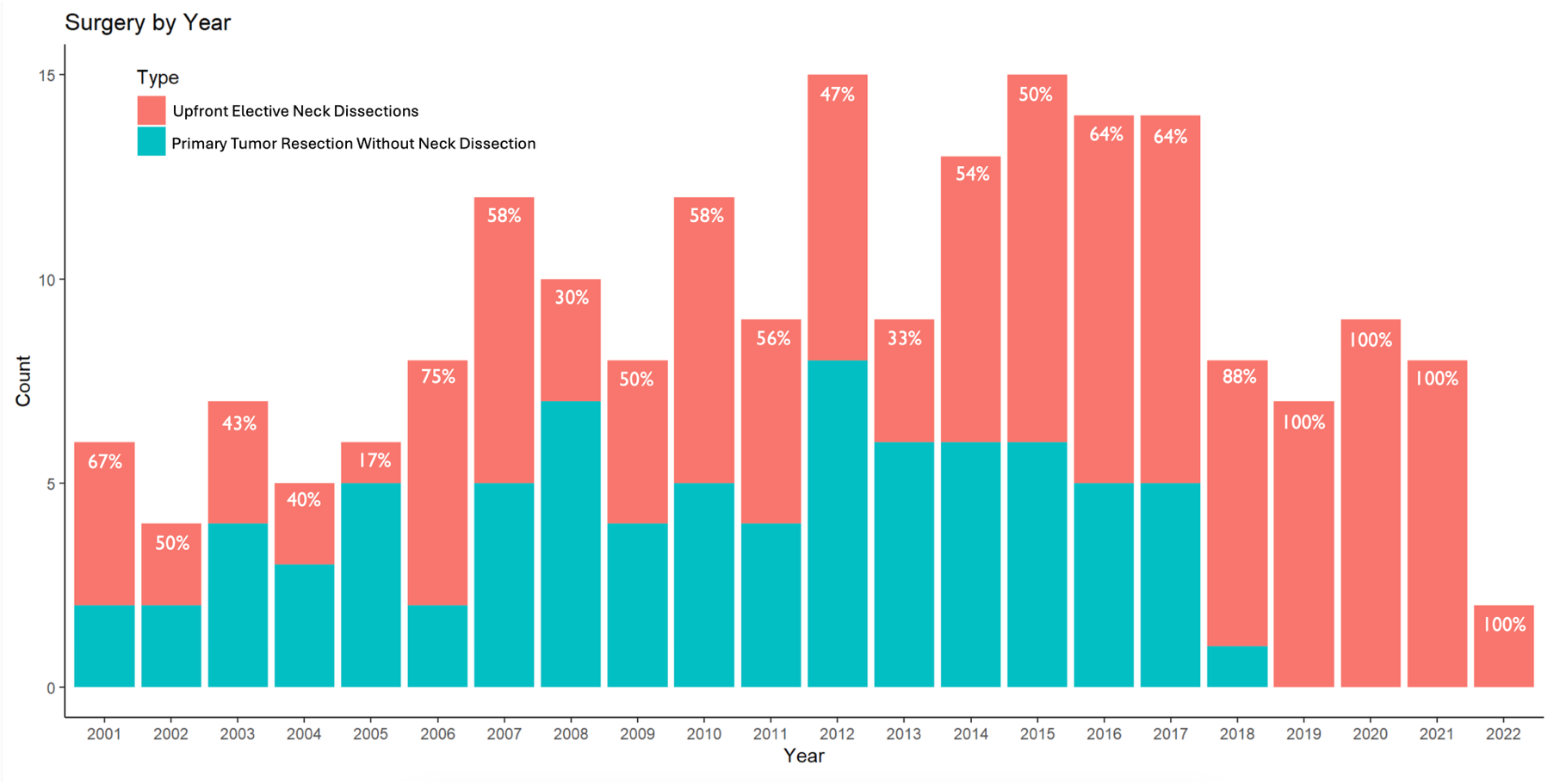

Supplement: Supplementary file 1 — Figure S1: Kaplan–Meier curves for 5‐year overall survival stratified by neck dissection. (A) total cohort (N = 200), (B) patients with primary tumor DOI ≥ 4 mm (N = 108), (C) tumor DOI ≥ 3 mm (N = 146), and (D) tumor DOI ≥ 2 mm (N = 167). Figure S2: Proportion of elective neck dissection by year of surgery. [file HED-48-339-s001.docx]
